# Supplementary material for: Photoacoustic Properties of Polypyrrole Nanoparticles
Source: Nanomaterials (Basel). 2021 Sep 21;11(9):2457. doi: 10.3390/nano11092457 (PMC8470055; doi:10.3390/nano11092457)
Supplement: Supplementary file 1 [file nanomaterials-11-02457-s001.zip › nanomaterials-1356307.pdf]

nanomaterials

Electronic Supplementary Information

Photoacoustic Properties of Polypyrrole Nanoparticles

Peter Keša [1], Monika Paúrová [2], Michal Babič [2], Tomáš Heizer [1], Petr Matouš [1], Karolína Turnovcová [3], Dana Mareková [3,4], Luděk Šefc [1] and Vít Herynek [1,*]

[1] Center for Advanced Preclinical Imaging (CAPI), First Faculty of Medicine, Charles University, Prague, 120 00, Czech Republic; peter.kesa@lf1.cuni.cz (P.K.); tomas.heizer@lf1.cuni.cz (T.H.); petr.matous@lf1.cuni.cz (P.M.); sefc@cesnet.cz (L.Š.)
[2] Institute of Macromolecular Chemistry, Czech Academy of Science, Prague, 162 06, Czech Republic; paurova@imc.cas.cz (M.P.); babic@imc.cas.cz (M.B.)
[3] Institute of Experimental Medicine, Czech Academy of Science, Prague, 142 20, Czech Republic; karolina.turnovcova@biomed.cas.cz (K.T.); marekova@biomed.cas.cz (D.M.)
[4] Second Faculty of Medicine, Charles University, Prague, 150 06, Czech Republic
* Correspondence: vit.herynek@lf1.cuni.cz; Tel.: +420-22496-5993

Experimental setup of in vitro photoacoustic measurements

Aliquots of the suspensions were injected into silicon tubes (inner/outer diameter 0.5/2.5 mm), adjusted into a phantom, and submerged in bubble-free water. The phantom was then scanned using a preclinical bimodal imaging platform Vevo 3100/LAZR-X (Fujifilm VisualSonics, Amsterdam, Netherlands) combining high frequency ultrasound and photoacoustics imaging as shown schematically in Figure S1. Figure S1a shows a schematic cross-section of a silicon tube filled with the suspension (nanoparticles PPyF4, concentration 1 mg/mL, excitation 800 nm), Figure S1b is an ultrasound image of the tube with pronounced signal from the front wall of the tube, weaker signal from the inner surfaces and very weak signal reflected from the back wall. Figure S1c shows photoacoustic signal produced by the nanoparticles injected in the tube. Evaluated area of interest is marked in both Figure S1b,c images.

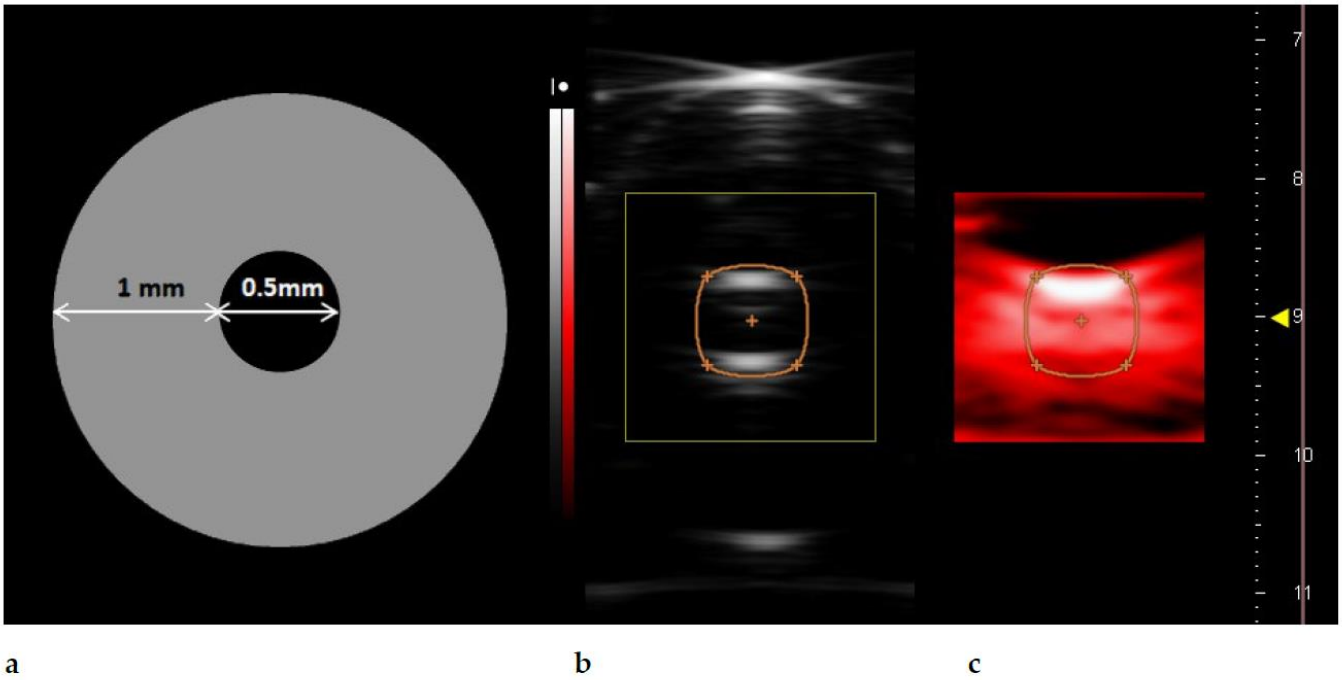

Figure S1. Experimental setup for in vitro measurements. A schematic cross-section of a silicon tube filled with the PPy-nanoparticle suspension (**a**), an ultrasound image of the tube with pronounced signal from the front wall of the tube, weaker signal from the inner surfaces and very weak signal reflected from the back wall (**b**), and a photoacoustic signal produced by the nanoparticles injected in the tube (**c**).

In vivo experimental setup

The anesthetized mouse was positioned on the heated working table in a supine position and conductively connected by four electrodes to monitor ECG and breathing. Anesthesia was maintained by passive inhalation of isoflurane (1–2%) in air. The ultrasound transducer equipped with a jacket with two (14 mm) optical fiber bundles was adjusted above the animal to achieve the best parasternal long axis view of heart's left ventricle, aorta, left and right atriums. The distance between transducer surface and animal skin (5 mm) was filled with a bubble free transparent ultrasound gel. The table with the animal and the probe was closed in a black box impervious to light to avoid parasitic excitation.

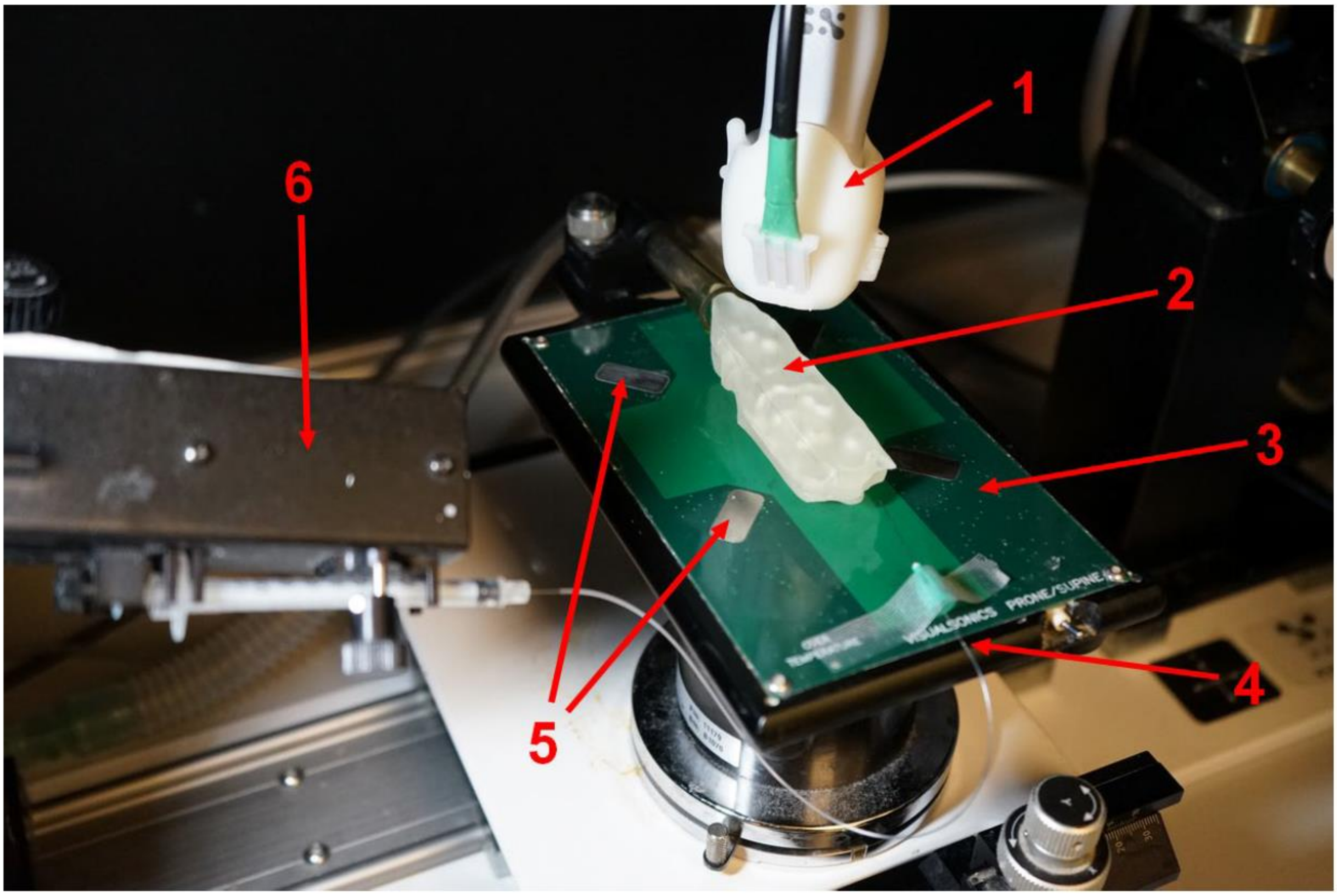

Figure S2. Experimental setup for *in vivo* measurements. 1a jacket with an ultrasound probe and two optical bundles for laser excitation, 2—a phantom representing the mouse, 3—a heated table for positioning the animal, 4—a catheter for nanoparticle application, 5—electrodes for ECG, 6—an infusion pump.

Cell Proliferation in the presence of the nanoparticles

The synthesized polypyrrole (PPy) nanoparticles PPyF3 prepared by oxidation of Py monomer (1.0 g, 14.72 mmol) with $FeCl_3$ (10 mL solution, *n*Py:*n*$FeCl_3$, 1:2.3, 9.15 g, 33.85 mmol) in the presence of PVP solution of specific concentration (160 mL; 1 wt% respectively) were coated to improve their biocompatibility for future in vivo applications. Three different coatings (poly-L-lysine (Mw ~70000, L3), Pluronic F-127-Plu3, and deblock-DB3) were used for coating.

The proliferation of C6 cells in the presence of both coated and uncoated PPyF3 particles was tested using the xCELLigence® RTCA DP instrument (ACEA Biosciences, San Diego, CA, USA). The method is based on the measurement of electrical impedance on a special 16-well microplate (E-Plate) equipped with gold electrodes at the bottom. As the cells grow, the impedance increases (adherent cells impede the flow of charge carriers in the cultivation media, which serves as electrolyte. The actual impedance depends on cell shape and size, the number of cells and the attachment quality (https://www.aceabio.com/products/rtca-dp/ 11 September 2021). Impedance is expressed as the Cell Index (dimensionless parameter).

Fifty 50 µL of cultivation media was added to each well and the background impedance measured. Next, C6 cells (10,000 cells per well) were seeded and left to attach for 30 min. During the log phase (approximately 2 h after the beginning of the experiment), the nanoparticles were added to achieve a final concentration of 1, 0.5, 0.25 mg/mL in the

well. Cells were then cultivated under standard cultivation conditions. Impedance was recorded every 15 min for 48 h. Each experiment was performed in doublets.

Both coated and uncoated PPyF3 nanoparticles transiently slowed down C6 cell proliferation at all tested concentrations (see ESI, Figure S2). In the long term, cells in the presence of low concentration (0.25 mg/mL or 0.5 mg/mL) of uncoated and L3-coated nanoparticles recovered and started to proliferate, while higher concentration (1 mg/mL) led to impairment of proliferation (see ESI, Figure S2 a,b). Plu3 and DB3 coatings turned out to be unsuitable; cell proliferation decreased at all concentrations of Plu3-coated nanoparticles, and at 0.5 and 1 mg/mL concentrations of DB3-coated PPyF3 nanoparticles (ESI, Figure S2 c,d).

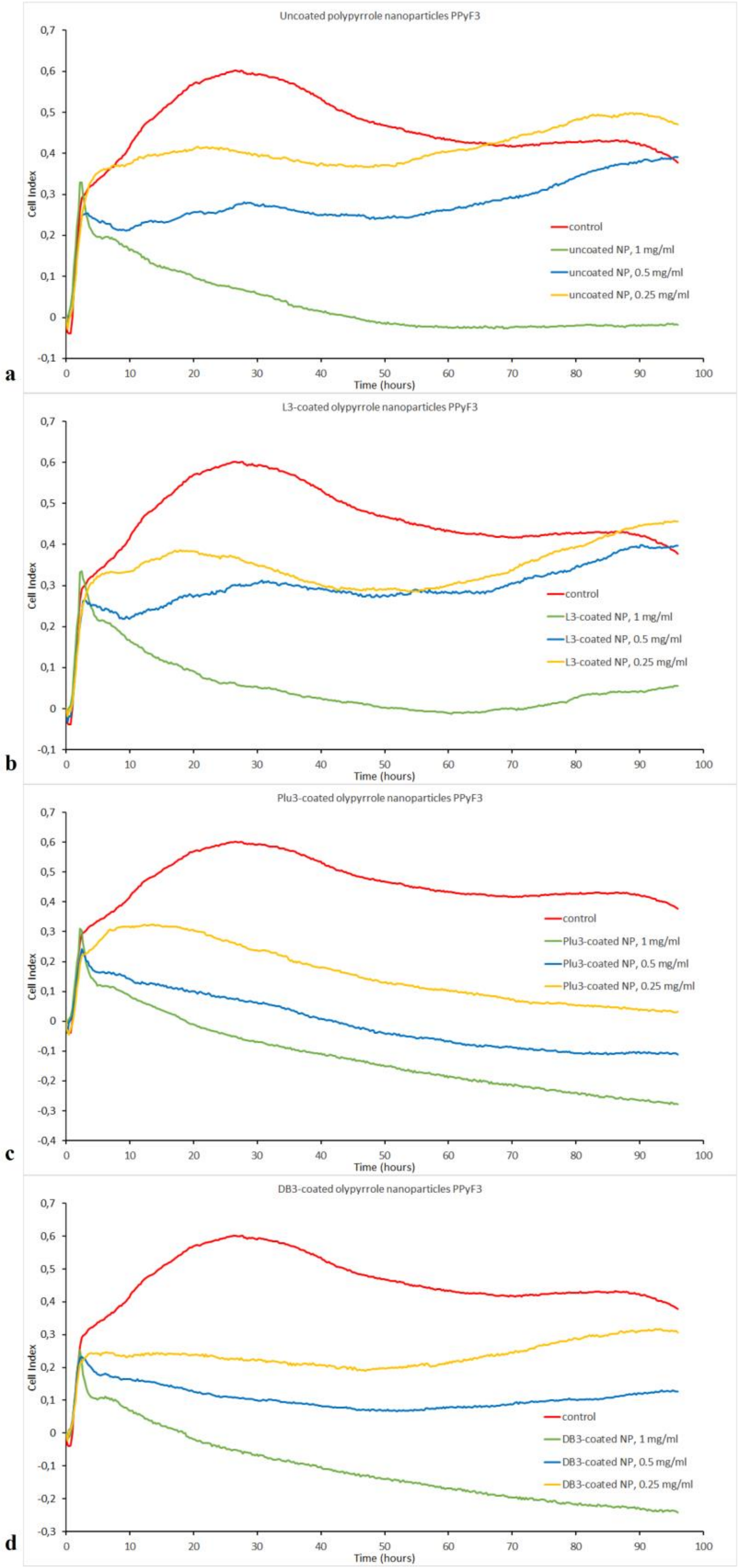

Figure S3. Cell Index corresponding to proliferation activity of C6 cells in the presence of uncoated (**a**), L3 coated (**b**), Plu3 coated (**c**), and DB3 coated (**d**) PPyF3 particles. Proliferation of the control sample (with no nanoparticles added) was inserted into all graphs (red line).

In Vivo Experiment

The PPyF4 nanoparticles were injected into the tail vein and the photoacoustic signal was acquired at 800 nm. Signal evolution before and after nanoparticle application is shown in Figure S2. The intense photoacoustic signal was clearly visible in the anterior wall of the mouse heart immediately after NPs injection, reaching its maximum approximately 5 s post injection. Then the signal slowly decreased. PA signal detected at 800 nm reached the reference value approximately 8 minutes after application.

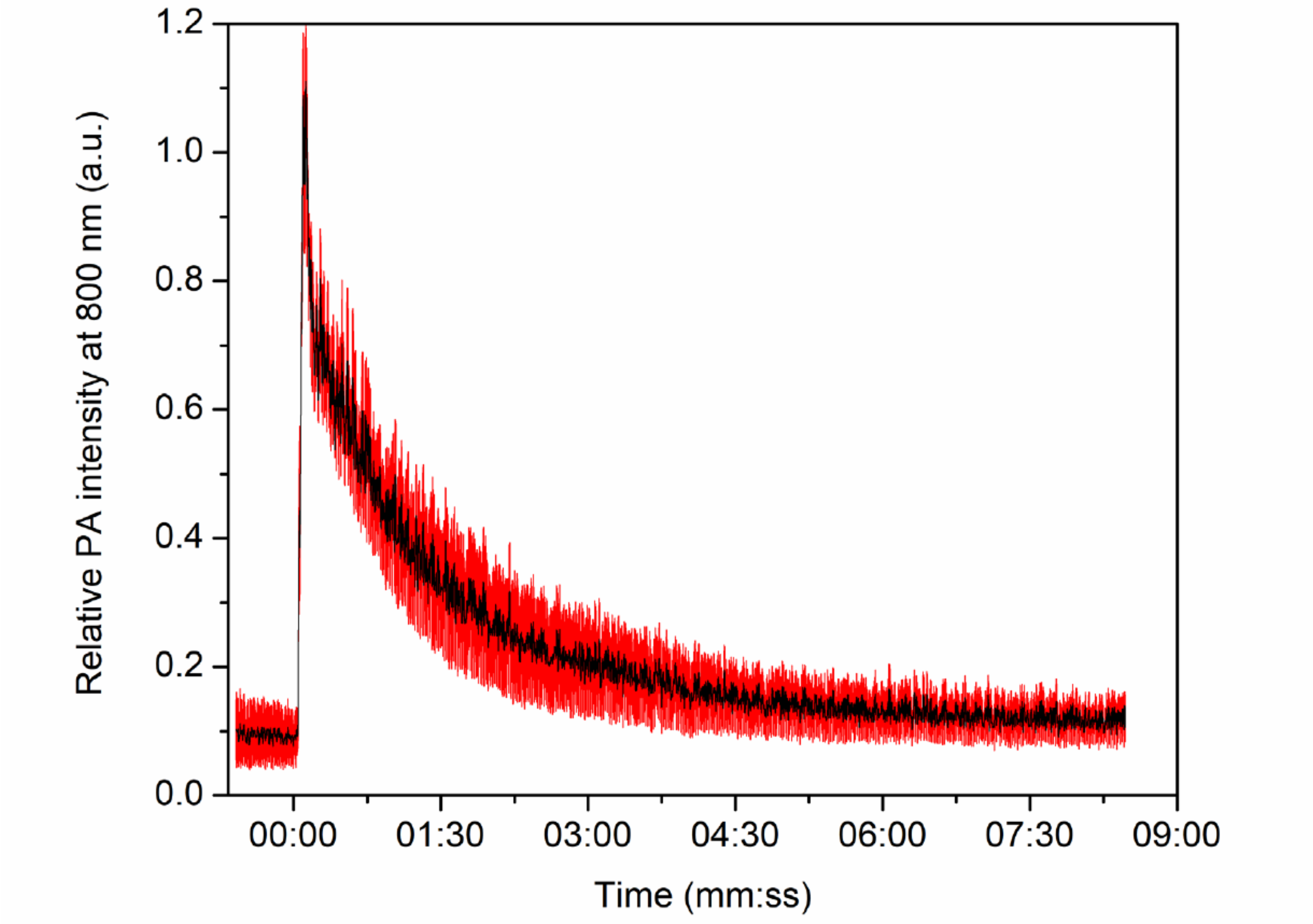

Figure S4. A photoacoustic signal detected at 800 nm excitation in the anterior heart wall of a mouse before and after intravenous application of PPyF4 nanoparticles. Application was performed at time $t = 0$ s.

Comparison of polypyrrole nanoparticles and indocyanine green

Spectrum of PPy particles PPyF4 (concentration 1 mg/mL) was compared to the spectrum of ICG, which is commercially available and is broadly used as a photoacoustic contrast agent (Figure S5). Polypyrrole particles provided substantially higher signal in NIR region (680–970 nm) than ICG (data in the range of 1200–2000 nm are not shown, ICG provides no measurable signal in this range).

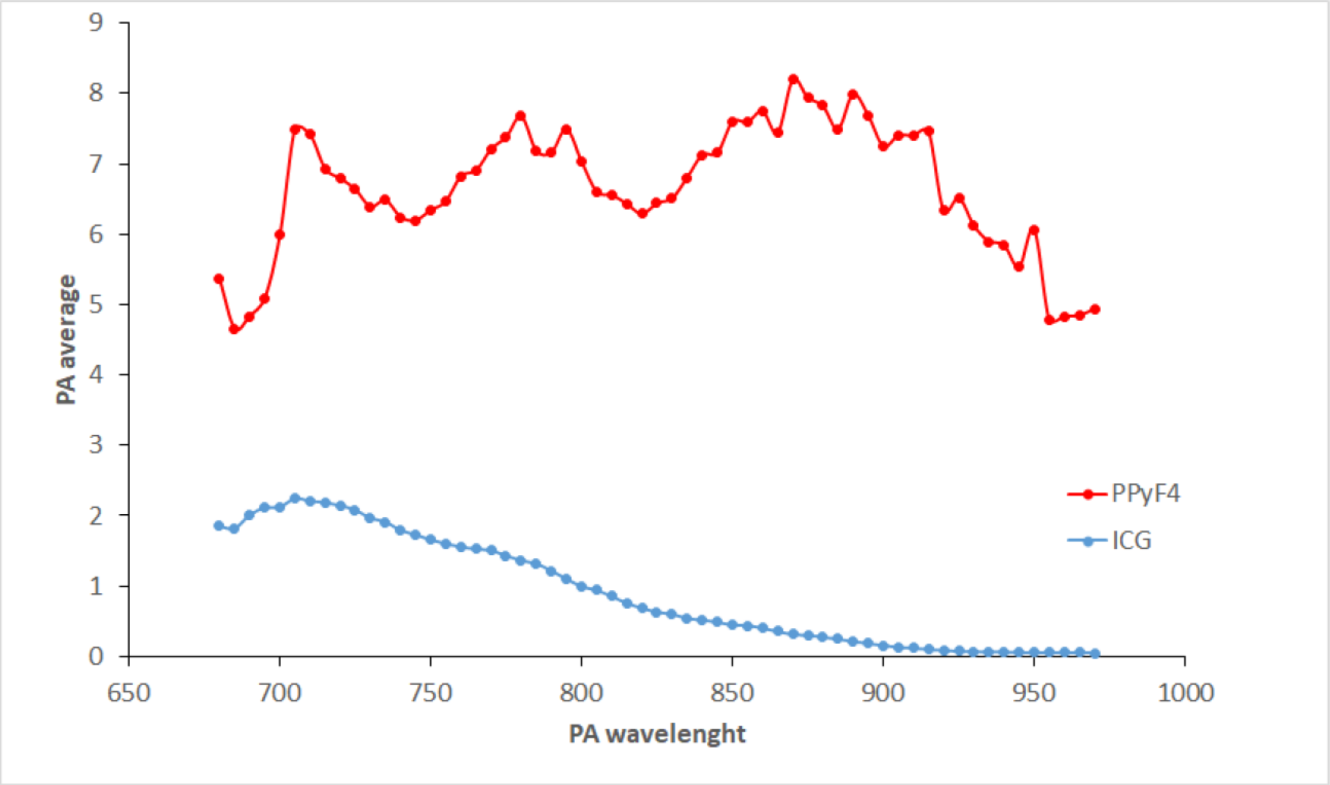

Figure S5. A photoacoustic spectra of polypyrrole nanoparticles PPyF4 (used also for in vivo experiment) and of indocyanine green ICG at the same concentration 1 mg/mL) in the range of 680–970 nm.
